# Ultrasound-Guided Conventional Versus Trans-isthmic Fine Needle Aspiration: Comparison of Post-biopsy Hematoma Rates and Patients’ Pain

**DOI:** 10.7759/cureus.31956

**Published:** 2022-11-28

**Authors:** Abdullah Yakupoğlu

**Affiliations:** 1 Department of Radiology, Memorial Şişli Hospital, Istanbul, TUR

**Keywords:** thyroid nodules, trans-isthmic biopsy, pain, hemorrhage, fine needle aspiration biopsy

## Abstract

Objective

Fine needle aspiration (FNA) is an invasive procedure; however, it is the simplest and safest method of identifying thyroid nodules that is well tolerated by patients. The most common complication is minor bleeding and pain. The objective of this study was to evaluate pain and complications among patients with thyroid nodules undergoing ultrasound (US)-guided FNA procedures.

Materials and methods

A total of 757 patients who underwent US-guided biopsy of the thyroid nodule in our institution between January 2017 and July 2022 were reviewed retrospectively. Demographic characteristics, US features, nodule depth and size, developing hematoma dimensions, and visual analog scale (VAS) scores during the procedure and at the 30th minute after the procedure were recorded in all cases.

Results

Overall, 272 (206 female and 66 male) patients who underwent US-guided conventional FNA (US-CFNA) procedure and 485 (361 female and 124 males) patients who underwent US-guided trans-isthmic FNA (US-TIFNA) were included. There was no significant difference between the two groups in terms of gender or age (p=0.691 and p=0.539, respectively). Although the mean nodule size of the US-TIFNA group was larger than that of the US-CFNA group, there was no statistically significant difference between the groups (p=0.137). There was a statistically significant difference between the two groups regarding the mean pain score during the procedures and 30 minutes after the procedure (p=0.032 and p=0.001, respectively). A total of 18 patients had procedural bleeding complications. While hematoma was detected in 11 (4.04%) patients who underwent the US-CFNA procedure, it was detected in seven (1.44%) patients who underwent the TIFNA procedure (p=0.024).

Conclusion

We compared the incidence of hematoma and the comfort of patients during and after the procedure between the US-TIFNA procedure and the US-CFNA procedure. The frequency of hematoma was higher in US-CFNA patients during and after the biopsy, and it was statistically significant. Moreover, the evaluation of patients’ pain levels has shown that the US-TIFNA procedure resulted in significant patient satisfaction.

## Introduction

Fine needle aspiration (FNA) biopsy is an invasive procedure; however, it is the simplest and safest method of identifying the nature of thyroid nodules that is well tolerated by patients [1,2]. FNA is a standard initial diagnostic tool for thyroid nodules, and it has a low complication rate [2-5]. The increased use of ultrasound (US) during FNA procedures has significantly reduced the risk of procedural complications [6,7]. Despite all advantages of US, bleeding is still the most common complication during conventional FNA procedures [8]. The glandular branches of the superior thyroid artery anastomose with the branches of the inferior thyroid artery, forming a vascular network that covers the surface of the entire thyroid gland. This vascular network that supplies the thyroid gland is open to injury during conventional FNA procedures. Although more thyroid tissue is traversed during the trans-isthmic FNA (TIFNA) procedure, it is less likely to encounter significant large-scale vascular structures. The isthmus is the region of the thyroid gland that connects both lobes of the thyroid gland, which is the most superficially and partially vascularized. Considering the vascular anatomy of the thyroid gland, it is likely that trans-isthmic interventions will cause less vascular injury. Although more thyroid tissue is traversed during the trans-isthmic FNA procedure, it is less likely to encounter significant large-scale vascular structures. In addition to the development of hematoma during FNA procedures, another important factor is how well patients tolerate the procedure. Significant differences in pain levels in patients undergoing US-guided thyroid biopsy have been reported in previous studies [9]. Therefore, we hypothesized that the trans-isthmic approach might be more appropriate to avoid possible vascular injury. Therefore, the aim of this study was to evaluate pain and hemorrhagic complications among patients with thyroid nodules undergoing US-guided FNA procedures.

## Materials and methods

Study population

A total of 757 patients who underwent US-guided biopsy of the thyroid nodule in our institution between January 2017 and July 2022 were reviewed retrospectively. All data of the patients were obtained from the hospital registry system. Patients with missing clinical sign/symptom records and post-procedure US scans were excluded from the study. This retrospective study was approved by the Ethics Review Board of our institution. Written informed consent was obtained from all patients before performing the procedures.

US-guided conventional FNA (US-CFNA) and trans-isthmic FNA (US-TIFNA) procedures

**Figure 1:**
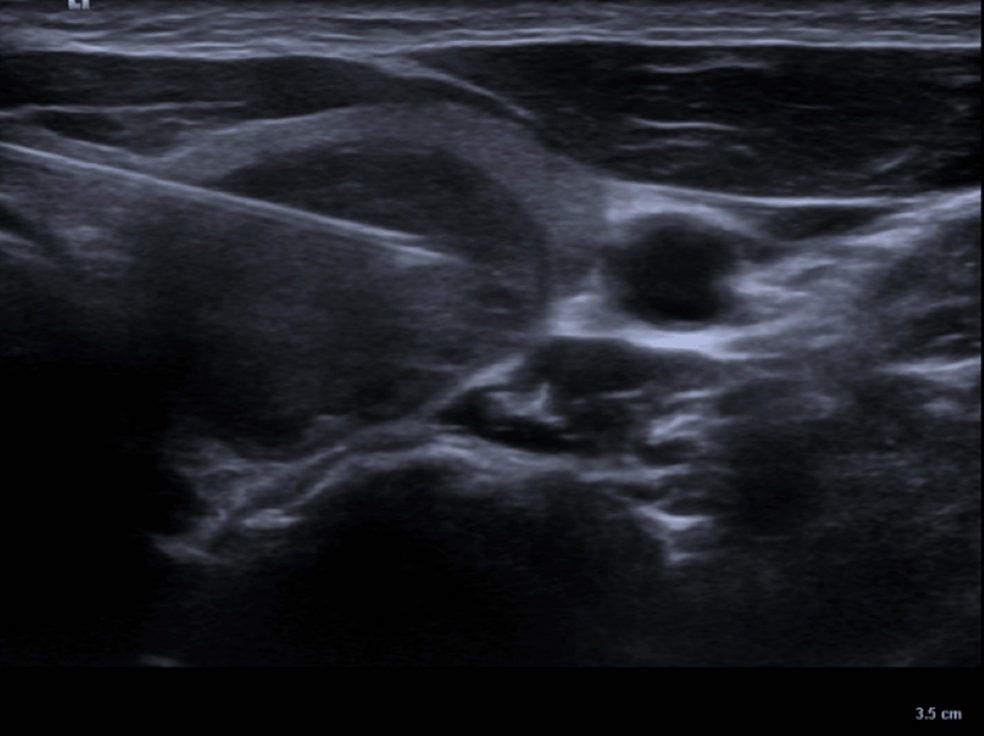
Biopsy of the lesion with trans-isthmic access.

All biopsy procedures were performed by an interventional radiologist with 20 years of experience, after a thorough review of relevant imaging studies. Patients taking aspirin or anticoagulants prior to the procedure were asked to discontinue their medication according to the recommendations of the latest guidelines [10]. All procedures were performed using a high-resolution ultrasound device (Acuson Juniper, Siemens Healthineers, Erlangen, Germany) with a 9.4-12.3 MHz linear transducer probe. To prevent vascular injury and hematoma formation, vascular mapping of lesions was performed before the biopsy. The US-CFNA procedure was performed using a 10-mL plastic syringe attached to a 22-gauge needle by entering from the lesion site medial to the sternocleidomastoid muscle under ultrasound guidance. The US-TIFNA procedure was performed using a needle and syringe of the same size by advancing the needle from the thyroid isthmus to the target nodule with a trans-isthmic approach under US guidance (Figure 1).

Demographic characteristics, US features, nodule localization and size, biopsy complications, developing hematoma dimensions, and visual analog scale (VAS) scores during the procedure and at the 30th minute after the procedure were recorded in all cases.

Statistical procedures were performed using a commercially available software package (Statistical Package for the Social Sciences (SPSS) version 19.0, IBM SPSS Statistics, Armonk, NY, USA). We analyzed and compared parameters including pain score and hemorrhagic complications between the US-CFNA and US-TIFNA groups. Comparison between the groups was made using an independent sample t-test and chi-square test. Probabilities of less than 0.05 were considered significant.

## Results

A total of 757 patients who underwent thyroid lesion biopsy were included in our study (272 patients underwent US-CFNA, and 485 patients underwent US-TIFNA). Of all patients, 567 (74.9%) were female and 190 (25.1%) were male. The demographic data of the patients and the size, depth, and US characteristics of the thyroid nodules are summarized in Table 1. There was no significant difference between the two groups in terms of gender or age (p=0.691 and p=0.539, respectively). Although the mean nodule size of the US-TIFNA group was larger than that of the US-CFNA group, there was no statistically significant difference between the groups (p=0.137) (Table 1).

**Table 1:** Patient demographic data and ultrasound findings. US-CFNA: ultrasound-guided conventional fine needle aspiration, US-TIFNA: ultrasound-guided trans-isthmic fine needle aspiration, mm: millimeter

	US-CFNA (n=272) (%)	US-TIFNA (n=485) (%)	p value
Age (years)	50.44±14.28	49.82±12.62	0.539
Sex			
Male	66 (24.3)	124 (25.6)	0.691
Female	206 (75.7)	361 (74.4)	
Size (mm)	18.4±13.03	19.8±10.8	0.137
Depth (mm)	9.93±4.36	9.39±4.27	0.096
Composition			0.480
Solid	192 (70.6)	322 (66.4)	
Semisolid	76 (27.9)	156 (32.2)	
Cystic	4 (1.5)	7 (1.4)	
Echogenicity			
Isoechoic	24 (8.8)	57 (11.8)	0.597
Hypoechoic	176 (64.7)	295 (60.8)	
Anechoic-cystic	4 (1.5)	7 (1.4)	
Mixed echoic	68 (25)	126 (26)	

While the mean VAS score of patients who underwent the US-CFNA procedure was 2.04±0.72 during the procedure and 1.15±0.6 at the 30th minute after the procedure, the mean VAS score of patients who underwent the US-TIFNA procedure was 1.92±0.76 during the procedure and 1.02±0.27 at the 30th minute. There was a statistically significant difference between the two groups regarding the mean pain score during the procedures and 30 minutes after the procedure (p=0.032 and p=0.001, respectively) (Table 2). A total of 18 patients had procedural bleeding complications. While hematoma was detected in 11 (4.04%) patients who underwent the CFNA procedure, it was detected in seven (1.44%) patients who underwent the TIFNA procedure (p=0.024) (Table 2). The mean hematoma size of patients who underwent the US-FNA procedure was 2.42±1.39 (range: 1.5-6.1) mm. The mean bleeding size in US-TIFNA patients was 1.93±0.44 (range: 1.4-2.7) mm (p=0.406).

**Table 2:** Comparison of the indicated parameters between the US-CFNA and US-TIFNA groups. US-CFNA: ultrasound-guided conventional fine needle aspiration, US-TIFNA: ultrasound-guided trans-isthmic fine needle aspiration, mm: millimeter

	US-CFNA (n=272)	US-TIFNA (n=485)	p value
Pain score			
During biopsy	2.04±0.72	1.92±0.76	0.032
After 30 minutes	1.15±0.6	1.02±0.27	0.001
Hematoma (n (%))	11 (4.04%)	7 (1.44%)	0.024
Hematoma size (mm)	2.42±1.39 (range: 1.5-6.1)	1.93±0.44 (range: 1.4-2.7)	0.406

## Discussion

In this study, procedure-related pain and complication rates were evaluated between two different procedures of FNA biopsy (CFNA and TIFNA) in patients who underwent US-guided thyroid biopsy.

Although US-guided thyroid biopsy has enormous advantages in diagnosing thyroid nodules, there are still concerns about its safety [7,11]. Although major complications requiring hospitalization or surgical intervention such as pseudoaneurysms and arteriovenous fistulas are very rare in US-FNA procedures, hematoma is the most common vascular injury [6,7,11-14]. Various hematomas can be detected at intrathyroidal, subcapsular, and perithyroidal locations during thyroid intervention but are usually successfully treated with neck compression. In patients with persistent bleeding on post-procedure US evaluation, close follow-up is necessary to monitor the growth of the hematoma. No major complications were observed in both FNA procedures performed in this article.

There are many studies in the literature evaluating the incidence of hematoma in CFNA procedures performed on thyroid nodules. Polyzos and Anastasilakis reported the incidence of bleeding complications during and after the conventional FNA biopsy procedure as 1.9%-6.4% in a large systemic review study [11]. However, due to the increase in the resolution and image quality of US devices, the rates of bleeding complications during and after FNA procedures have decreased drastically. In 2017, Chae et al. reported 0.7% hematoma after US-guided conventional biopsy in 5,121 patients [6]. In our study, the incidence of hematoma after US-CFNA biopsy was 4.04%. Our results were higher than previous studies with US-guided FNA biopsies performed under conditions similar to ours [6,15]. The high incidence of bleeding in our study can be attributed to our sensitive bleeding criteria; any parenchymal edema or thin perithyroidal/perilesional soft tissue or fluid was considered as bleeding because we hypothesized that any minor bleeding could progress and cause serious complications. Despite our high bleeding criteria, bleeding complications were observed in only seven (1.44%) patients who underwent the US-TIFNA procedure, which is similar to previous studies. In addition, it is seen that it is statistically significant compared to US-CFNA biopsy in terms of bleeding complications during or after the procedure in our patients who underwent the US-TIFNA procedure (p=0.024).

Although FNA is a simple, safe, and well-accepted method, it is an invasive procedure, and mild pain or discomfort can be observed at the aspiration site. Few studies have investigated the difference in subjective experiences of patients undergoing FNA procedures. Pain intensity between 21 gauge and 23 gauge was evaluated in a group of patients who underwent conventional FNA biopsy. Although the mean VAS values ​​differed according to the thickness of the biopsy needle used, it was not statistically significant. In the study conducted by Jung et al., the mean VAS values ​​during the procedure were reported as 1.8±1.3 (range: 0-6) in the 21-gauge needle group and 1.4±1.1 (range: 0-5) in the 23-gauge needle group [16]. In our study, a 22-gauge needle was used in the US-CFNA group, and the mean VAS value was 2.04±0.72 (range: 0-5) during the procedure and 1.15±0.6 (range: 0-5) at the 30th minute after the procedure. These results are similar to previous studies. However, in the group of patients who underwent the US-TIFNA procedure, despite the use of a 22-gauge needle as in the US-CFNA group, a significant decrease was observed in the mean VAS scores during and after the procedure. In the US-TIFNA group, the mean VAS score was 1.92±0.76 (range: 0-5) during the procedure and 1.02±0.27 (range: 0-4) at the 30th minute after the procedure. Significant differences were found between the two procedures in terms of mean VAS scores (p=0.032 and p=0.001, respectively).

There are several limitations to our study. First is the retrospective study design, which may reveal an inherent bias in patient selection. However, all patients were enrolled in the study consecutively. Second, the choice of the biopsy procedure was made according to the operator’s preference, and it was the source of selection bias. Finally, the single-center design of the study may make it non-generalizable. Future prospective multicenter studies may reduce these limitations.

## Conclusions

We compared the incidence of hematoma and the comfort of patients during and after the US-TIFNA procedure and the US-CFNA procedure. The frequency of hematoma was higher in US-CFNA patients during and after the biopsy, and it was statistically significant. Considering the vascular anatomy covering the surface of the thyroid gland, we thought that there may be a risk of damage to these structures during the conventional FNA procedure. Moreover, the evaluation of patients’ pain levels has shown that the US-TIFNA procedure resulted in significant patient satisfaction.
